# High Speed Imaging of Cavitation around Dental Ultrasonic Scaler Tips

**DOI:** 10.1371/journal.pone.0149804

**Published:** 2016-03-02

**Authors:** Nina Vyas, Emilia Pecheva, Hamid Dehghani, Rachel L. Sammons, Qianxi X. Wang, David M. Leppinen, A. Damien Walmsley

**Affiliations:** 1 Physical Sciences of Imaging for Biomedical Sciences (PSIBS) Doctoral Training Centre, College of Engineering & Physical Sciences, University of Birmingham, Birmingham, B15 2TT, United Kingdom; 2 School of Dentistry, College of Medical and Dental Sciences, University of Birmingham, St Chad's Queensway, Birmingham, B4 6NN, United Kingdom; 3 School of Computer Science, University of Birmingham, Edgbaston, Birmingham, B15 2TT, United Kingdom; 4 School of Mathematics, University of Birmingham, Edgbaston, Birmingham, B15 2TT, United Kingdom; University of Zurich, SWITZERLAND

## Abstract

Cavitation occurs around dental ultrasonic scalers, which are used clinically for removing dental biofilm and calculus. However it is not known if this contributes to the cleaning process. Characterisation of the cavitation around ultrasonic scalers will assist in assessing its contribution and in developing new clinical devices for removing biofilm with cavitation. The aim is to use high speed camera imaging to quantify cavitation patterns around an ultrasonic scaler. A Satelec ultrasonic scaler operating at 29 kHz with three different shaped tips has been studied at medium and high operating power using high speed imaging at 15,000, 90,000 and 250,000 frames per second. The tip displacement has been recorded using scanning laser vibrometry. Cavitation occurs at the free end of the tip and increases with power while the area and width of the cavitation cloud varies for different shaped tips. The cavitation starts at the antinodes, with little or no cavitation at the node. High speed image sequences combined with scanning laser vibrometry show individual microbubbles imploding and bubble clouds lifting and moving away from the ultrasonic scaler tip, with larger tip displacement causing more cavitation.

## Introduction

Ultrasonic scalers are used for debridement of dental plaque and calculus from tooth crown and root surfaces during routine periodontal therapy [1]. Their primary method of cleaning is through contact of the vibrating metal tip to the surface to be cleaned. However, it has been shown in vitro that cavitation occurs in the cooling water around ultrasonic scaler tips which may also be aiding in the cleaning process [2].

Cavitation is the formation, growth and collapse of microscopic bubbles in liquids when the local pressure falls below the saturated vapour pressure of the liquid [3,4]. The collapse of the bubbles is violent and can result in the generation of high velocities, pressures and temperatures. In ultrasonic scalers it may be helping to disrupt bacterial plaque and mineralised calculus [5]. Various physical cavitation effects are observed, including: formation of a re-entrant micro-jet when near a boundary such as a wall or other bubbles which can impact a surface at high velocity; generation of large amplitude shock waves; micro-streaming in the surrounding fluid—small oscillations of fluid elements; micro-streamers—cavitation bubbles travelling in ribbon-like formations towards nodes or antinodes; and bubble migration towards pressure nodes and antinodes due to Bjerknes forces [4,6–8].

Further investigation of cavitation occurring around ultrasonic scaler tips is required to determine its cleaning effectiveness and to aid in the development of cavitation-optimised ultrasonic scalers which could potentially lead to more efficient, non-contact removal of biofilm from tooth surfaces and dental implants. One method to investigate the spatial distribution and the degree of cavitation occurring around ultrasonic scaler tips is sonochemiluminescence (SCL) [2,3,9,10]. Studies using SCL have proven that cavitation can indeed occur around ultrasonic scalers, and that it does not happen along the whole length of the tip, but in clusters. However as it is an indirect measure, it does not show cavitation in real time, and quantitative information (e.g. bubble cloud size and microbubble diameter) cannot be obtained. Therefore there is little information about cavitation at the free end of the scaler tip. The end of the tip is the most clinically relevant part as it contacts the biofilm most often and is used when accessing difficult-to-reach areas such as periodontal pockets around the teeth.

High speed imaging has been used to successfully characterize cavitation activities in fields such as mechanical and biomedical engineering [11–13], and in dentistry for endodontics [14–16]. It can image the cavitation directly instead of imaging the indirect luminol luminescence, showing bubble activity in real time. In addition, the resolution is higher than SCL, allowing image analysis to be done on individual bubbles and clusters and allowing more detailed observation of the cavitation activity at the free end of the tip. Scanning laser vibrometry (SLV) has been used to make non-contact measurements of the oscillations of ultrasonic scaler tips. Combining data from these different techniques allows us to understand the link between scaler vibrations and occurrence of cavitation at the most clinically relevant part of ultrasonic scalers.

The aims of this study were to use high speed imaging, SLV and image analysis to investigate how cavitation at the free end of the tip varied with power and tip design, and to observe and provide quantitative data on various sono-physical phenomena occurring around ultrasonic scaler tips.

## Materials and Methods

### High Speed Imaging

**Fig 1:**
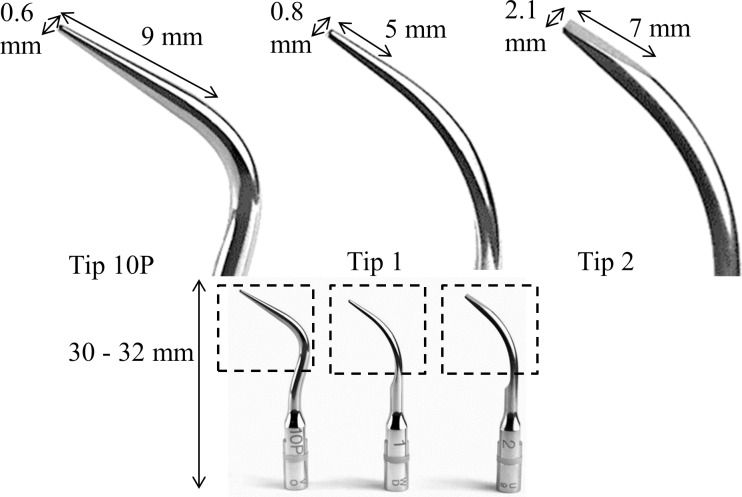
The three Satelec ultrasonic scaler tip designs imaged in this study. Tip 10P is the most pointed, tip 1 is pointed but tapers out towards the bend and tip 2 is flat with a wide cross-section.

A Satelec P5 Newtron Scaler (Acteon Group, USA) was used for all experiments in conjunction with Satelec tips 10P, 1 and 2. (Fig 1). High speed camera imaging was done with the scaler operating at Power 10 (medium) or Power 20 (maximum).

**Fig 2:**
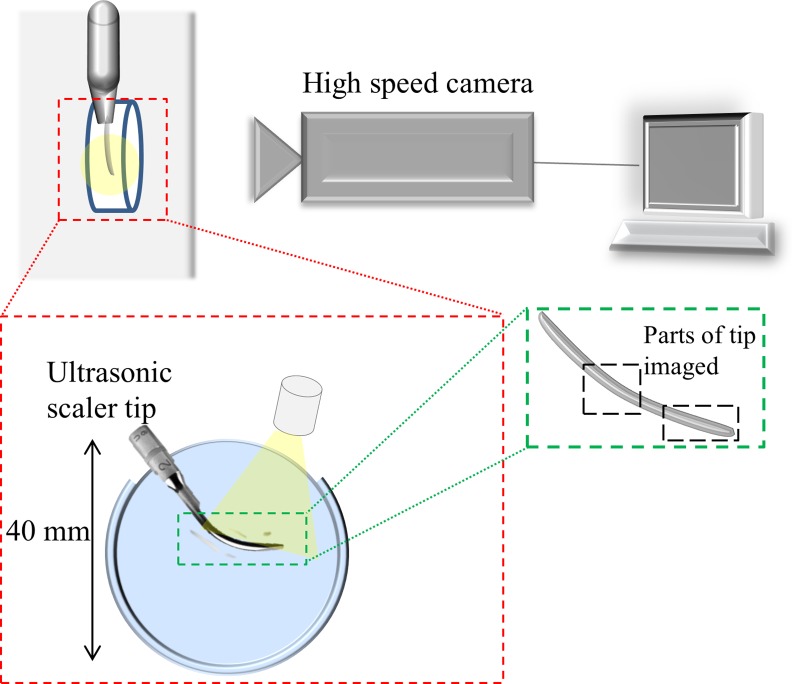
Schematic of the experimental setup for high speed imaging, showing the petri dish used for imaging and a view of the setup from above.

The flow rate of the cooling water flowing out of the scaler tip at the lowest flow setting is 0.11±0.01 ml/s. As it was difficult to image clearly with the water flow from the scaler, the tips were submerged in water with the cooling water turned off. A watertight container with transparent, flat sides was made for high speed imaging by attaching the sides of a 40 mm diameter petri dish, and cutting an opening at the top for inserting the ultrasonic scaler tip (Fig 2). Tips were submerged in 10 ml of distilled water at 20.5°C for imaging. The scaler tip under observation was clamped into place within the container, ensuring that no parts of the scaler body were touching the container.

Imaging was undertaken with a monochrome Photron Fastcam SA.1.1 (Photron, San Diego, CA, USA) attached to a zoom lens (Monozoom 7, Leica Microsystems UK Ltd) (Table 1). The size of each pixel was calculated from measurements of a 2 mm graticule with 10 μm markings. The scaler was illuminated for high speed imaging with a cold light source (KL 1500, Schott, Stafford, UK) placed either directly above the opening of the container or behind the container for imaging in bright field mode (Fig 2).

**Table 1:** Settings used during high speed imaging of cavitation around various parts of the ultrasonic scaler tips.

High speed imaging Experiment	Frames per second (fps)	Zoom lens settings
**Imaging free end of scaler tip**	90,000	x2 objective at x1 magnification
**Imaging individual bubbles at tip end**	250,000	x2 objective at x4 magnification
**Imaging bend of scaler tip**	15,000	x2 objective plus x3 tube extension at x1 magnification

### Image Analysis

Image segmentation was done via the trainable Weka Segmentation Plugin in Fiji (ImageJ, U. S. National Institutes of Health, Bethesda, Maryland, USA) [17,18]. SigmaPlot 12.3 (Systat Software Inc, San Jose, CA, USA) was used for analysis and graphing of the data. The Mann-Whitney U test was used for testing for statistical significance.

**Fig 3:**
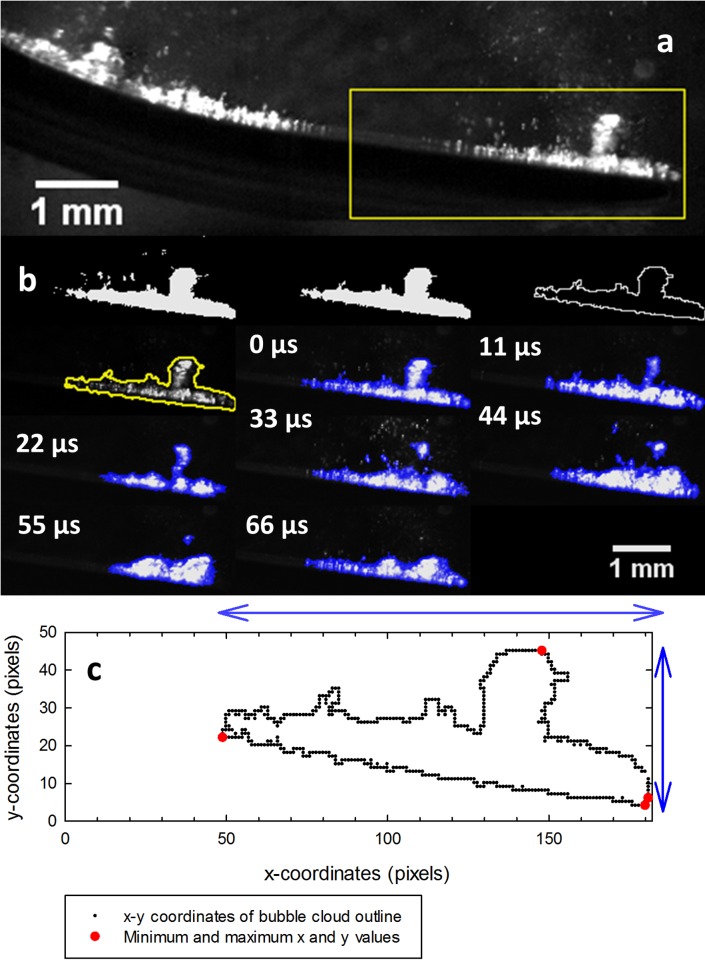
Segmentation and quantification process (illustrated with a frame from a video of tip 2 at power 20). **(a)** Raw image showing the region of interest selected for image analysis. **(b)** left to right: Thresholded segmented image of bubble cloud; image after removing any objects smaller than 20 pixels; binary outline of the segmented image; overlay of the segmented outline on top of the original bubble cloud to show the bubble cloud has been segmented accurately; sequential images showing the smaller bubble cloud lifting away from the main cloud. (Overlay of the original images and the thresholded images in blue) **(c)** graph illustrating how the width and the height of the bubble cloud was calculated from the minimum and maximum x and y coordinates.

Analysis was done using three video repeats for each measurement, with each video containing 940 images (totalling 2820 images per setting). The following steps were performed to segment and measure bubble clouds in the image sequences:

1. Each image was cropped to the same size (4 mm x 1.5 mm) to keep only the bubble cloud at the inner end of the tip (Fig 3A).
2. The trainable Weka segmentation plugin in Fiji was used to segment a whole image stack of 940 images. 200 images from each stack were used to create and train a classifier by manually choosing and classifying areas as background or as part of the bubble cloud on 3–4 of the images. The classifier was then applied to the whole stack to automatically segment the images.
3. Any objects smaller than 20 pixels were removed from the binary segmented images, to only keep bubbles that were part of (or had emerged from) the main bubble cloud (Fig 3B and 3C).
4. The area of each segmented bubble cloud was measured in Fiji using the Analyze Particles plugin. The width and the height of the cloud were measured by extracting the bubble coordinates (Fig 3D and 3F). The largest x-coordinate was subtracted from the smallest to give the width of the cloud and vice versa in the y direction to give the height. This data was plotted in box and whisker plots.

### Scanning laser vibrometry

The vibration analyses of the scaler tips were performed using a high frequency scanning vibrometer system (PSV 300-F/S, Polytech GmbH, Waldbronn, Germany), in conjunction with an He–Ne laser (λ = 632.8 nm, class 2). A frequency measurement range of 0–100 kHz was selected to allow detection of the fundamental frequency of the scaler (29 kHz, λ≈5 cm), as well as higher order harmonics. The SLV was set to perform fast Fourier transformation using 800 data points. The laser beam was focused at the tip free end and a number of equally spaced scan points were chosen along the tip length from the free end to the bend of the tip. The hand piece was clamped so that the outer front part of tip was vertical and clearly visible to the SLV camera. The maximum displacement of the tip at each scan point was measured and an average of 5 measurements were recorded for each tip at power 10 and 20. Each scan lasted approximately 10 s with an interval of 20 s between scans.

## Results

**Fig 4:**
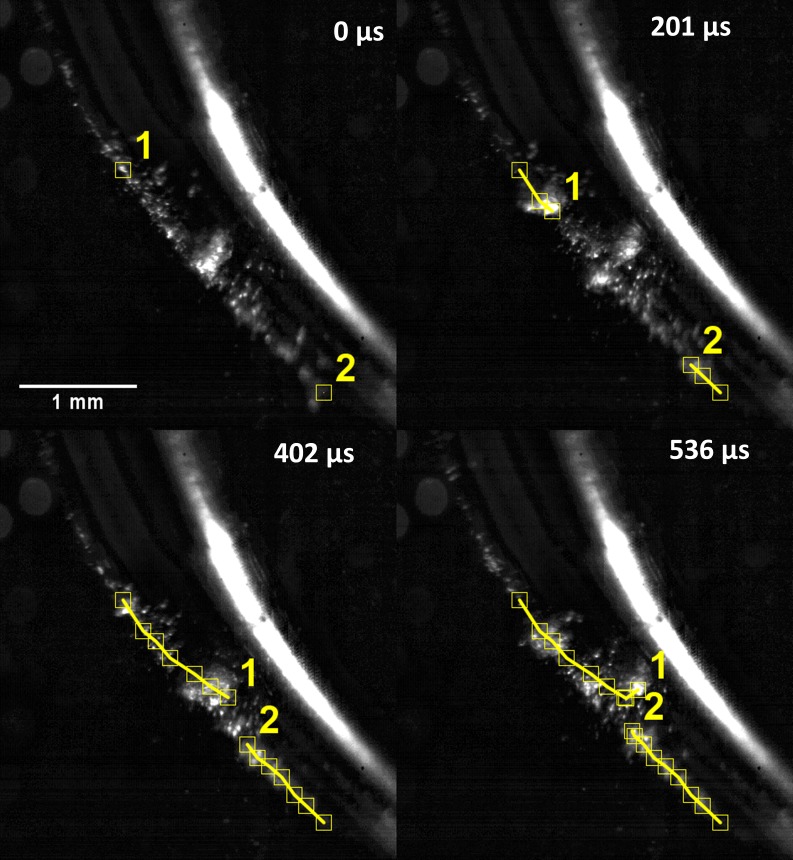
Still images from a high speed video of the bend of tip 1 at power 10 (refer to Fig 2 to see the part of the tip imaged). Two smaller bubble clusters have been tracked and labelled as they migrate towards a larger bubble cluster in the centre. Also see S1 Video.

Cavitation around the outer bend of tip 1 was imaged at 15,000 fps (Fig 4, S1 Video). Smaller bubble clusters can be seen migrating towards a larger bubble cluster in the centre. Manual tracking follows two of these clusters (labelled 1 and 2) approaching from opposite sides until they meet at the larger bubble cluster at the centre. This also occurred at the bend of tip 2 and tip 10P. The ultrasonic scaler completed nearly 6 oscillations between the frames shown in the figure.

**Fig 5:**
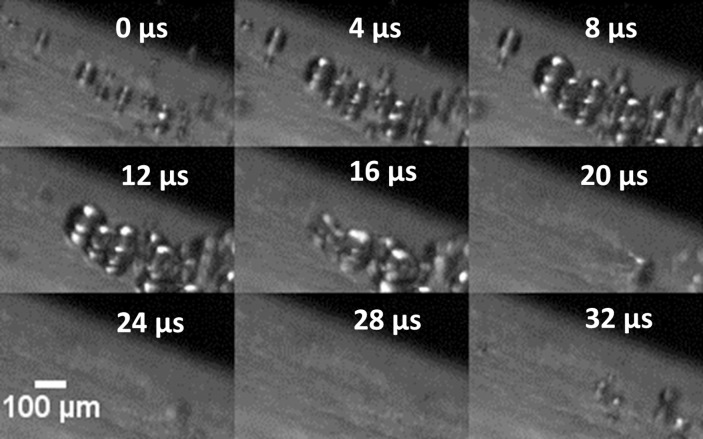
Still images from a high speed video of the inner part of the free end of tip 1 at power 10. Individual microbubbles are seen to grow and collapse on the scaler tip.

**Fig 6:**
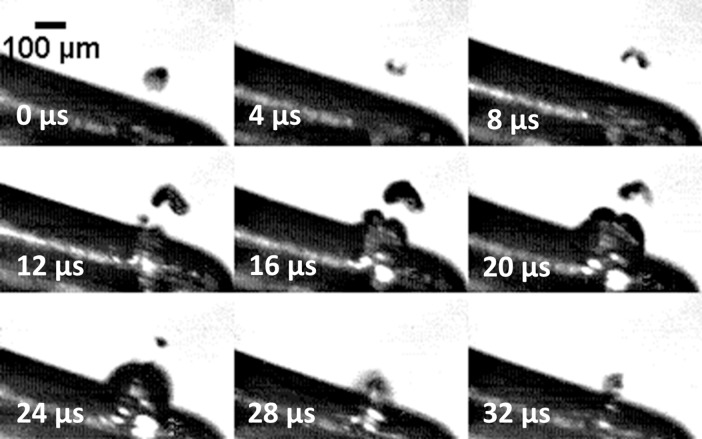
Still images from a high speed video of the inner part of the free end of tip 10P at power 10. A single microbubble can be seen to grow, split in two and collapse onto the scaler tip. Also see S2 Video.

High speed images at 250,000 fps show individual bubbles forming and collapsing at the inner end of tip 1 and tip 10P (Figs 5 and 6, S2 Video). On tip 1 the diameter of the microbubbles observed ranges from 35–150 μm. Fig 6 shows a distinct ‘v’ shape made by a bubble on tip 10P during the collapse phase.

As well as individual bubbles, entire bubble clouds at the tip were imaged on the three tips at 90,000 fps (Fig 3, S3,S4 and S5 Videos). Bubble lift-off was observed at power 20 for all tips where small bubble clouds emerged from the main cloud and were propelled away from the scaler tip into the surrounding water. This can be seen in Fig 3 where a cluster has lifted above the main cloud and detaches in the subsequent frames, and in S3, S4 and S5 Videos.

**Fig 7:**
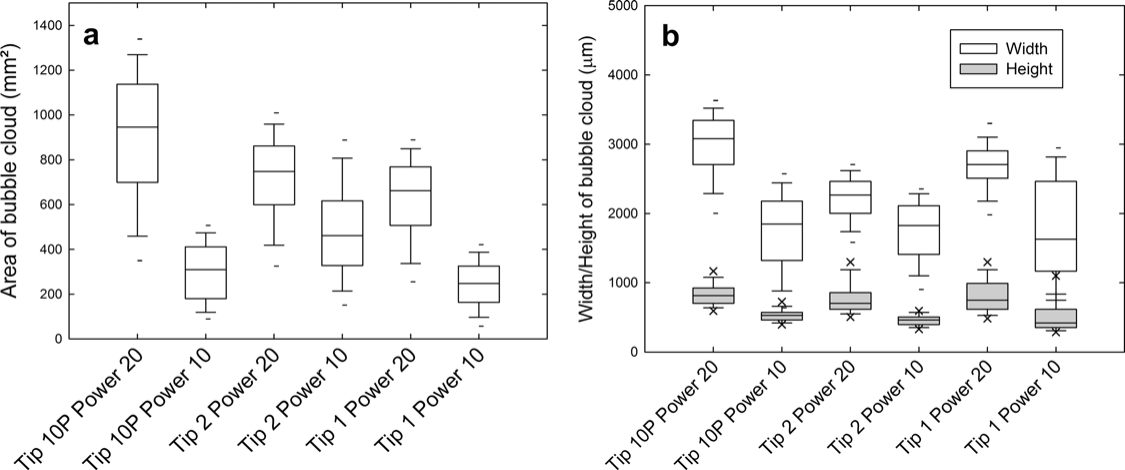
Box and whisker plots of (a) the area of the bubble clouds at the tip of three different ultrasonic scaler tips operating at medium and high (b) the height and width of the bubble cloud.

The box and whisker plots show only the outliers in the top 5^th^/95^th^ percentiles for clarity (Fig 7). The area of the cloud of cavitation bubbles at the tip was larger at the highest power setting compared to the medium power setting. Tip 10P at power 20 showed the most variation in area. The Mann-Whitney U test was used to compare the median values of each measurement setting. The difference in the areas between the same tip at different powers was statistically significant (*p<0*.*001*) for all three tips, and the difference between different tips at the same power was also statistically significant (*p<0*.*001*). Tip 2 showed the smallest area of cavitation out of the three tips measured.

The difference in the height and width of the bubble cloud at the tips was also statistically significant between the same tip at different powers (*p<0*.*001*). This was also valid for the difference between different tips at the same power for all except the following two combinations for which there was no statistically significant difference: tip 10P and tip 2, and tip 10P and tip 1 at power 10 (*p = 0*.*331* and *p = 0*.*151* respectively).

**Fig 8:**
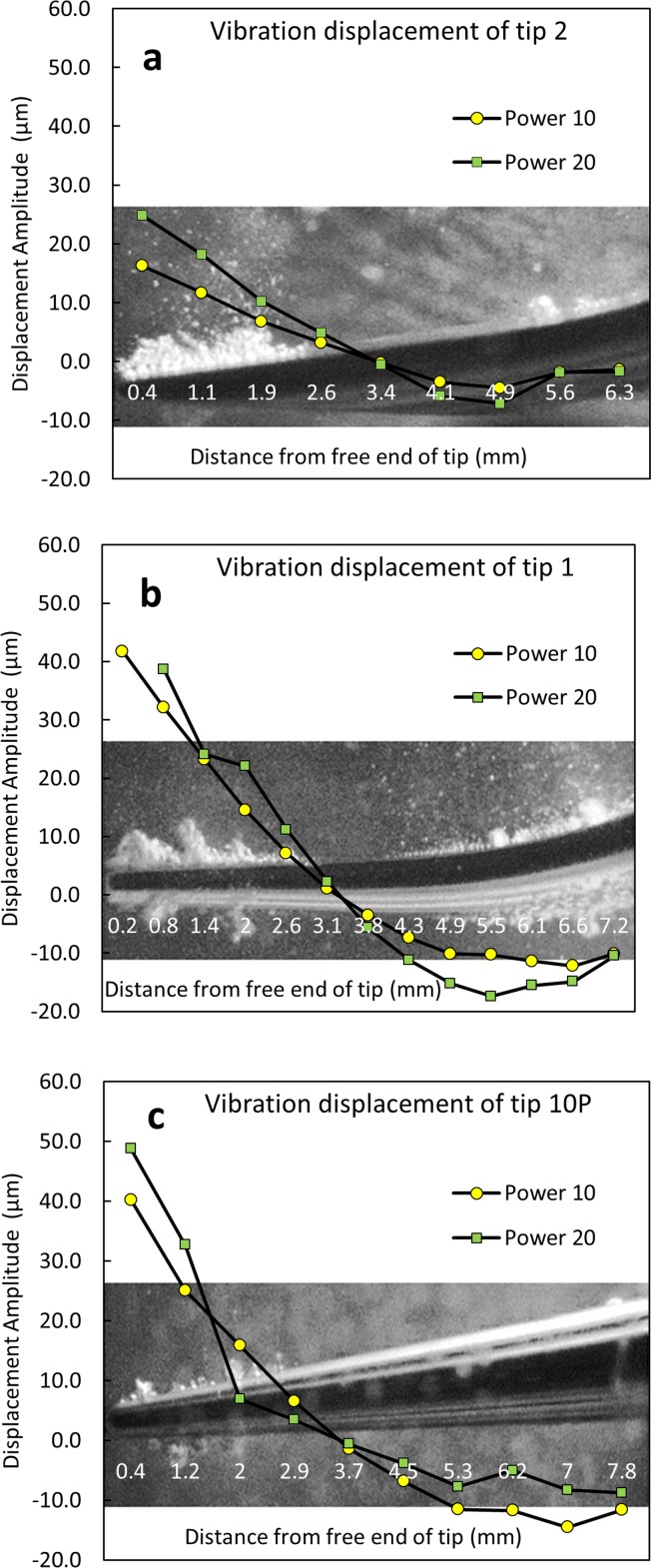
Displacement amplitudes of each of the three tips measured at medium power (power 10) and high power (power 20) using scanning laser vibrometry (SLV). The images overlaid on each graph are stills from high speed images of the corresponding tip taken at power 20 and are scaled in the x direction.

**Fig 9:**
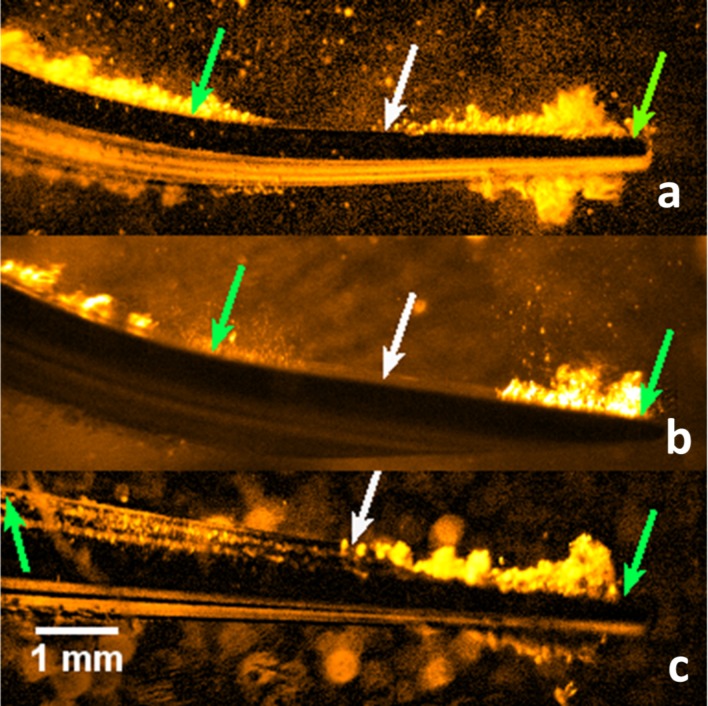
Contrast enhanced image stills from a high speed video of tip 1 (a), tip 2 (b) and tip 10P (c) at power 20, showing cavitation occurring around the free end and at the bend of the tips. Green arrows indicate points of largest tip vibration and white arrows indicate the point of least displacement. Also see videos 3, 4 & 5.

The tip displacement amplitude was greater at power 20 compared to power 10 (Fig 8). Tip 10P showed the largest displacement at the free end of the tip and tip 2 showed the smallest. However at the bend, tip 1 showed the largest displacement and tip 2 showed the smallest. In between these two antinodes there is a node (point of smallest displacement). Overlaying the high speed images with vibration data shows that cavitation happens at antinodes and does not happen at nodes (Fig 9).

## Discussion

Migration of bubbles towards a central bubble cluster was observed (Fig 4, S1 Video). This is a result of Bjerknes forces, which act on bubbles in a sound field and arise from the combination of the volume pulsations of the bubble and the acoustic pressure gradient [19]. At the bend of the tip in Fig 4, smaller bubbles join to form larger bubbles whilst moving along the tip. The larger bubbles come from both directions along the tip then join at a central point before dissolving. Eq 1 from Brennen et al. [4] shows that the resonant bubble radius is 697 μm (resonance occurs at 29 kHz).

$$\omega_{N}=\sqrt{\frac{1}{\rho R_{E}^{2}}\left\{3k(p_{\infty }-p_{v})+2(3k-1)\frac{\sigma }{R_{E}}\right\}}$$ (1)

*ω*_*N*_ is the natural frequency (*ω*_*N*_ = 29 kHz), *p*_∞_ is the pressure in the undisturbed liquid (*p*_∞_ = 100 kPa), *p*_v_ is the partial pressure of vapour of the bubble (*p*_v_ = 3 kPa), *k* is the polytropic index (*k* = 1.4), σ is the surface tension coefficient (*σ* = 0.07 Nm^-1^), *ρ* is the density of the liquid (*ρ* = 1000 kgm^-3^), and *R*_*E*_ is the bubble radius.

The radius of the large bubbles observed at the bend of the tip is ~190 ± 30 μm. As they are smaller than the resonance size, the primary Bjerknes force causes them to migrate up a pressure gradient (whilst bubbles larger than the resonance size would migrate down a pressure gradient) [19]. As the bubbles can be seen to be collecting at a point at the centre of the bend, we speculate that there is a standing wave and that the bubbles are collecting at a pressure antinode. We also speculate that the smaller bubbles travelling in ribbon-like structures are acoustic streamers, as described by Doinikov et al [20] and Lauterborn et al [6].

At the end of tip 10P a single bubble was seen to split into two bubbles and formed a V-shape (Fig 6, S2 Video). This is common during the formation of high-speed micro-jets when a cavitation bubble collapses near a boundary [21,22]. The bubble collapse happens much faster than the growth phase so the micro-jet cannot be visualised in this set of images taken at 250,000 fps. A frame rate of 1 million fps would show this. In this case the closest boundary was the ultrasonic scaler tip itself so the bubble collapsed onto the tip. The overall aim is to use cavitation from ultrasonic scaler tips to remove biofilm from teeth and dental implants, aiding in the cleaning process [3]. Therefore the bubbles must collapse onto the biofilm instead of on the scaler. The bubble clouds reached a height of approximately 0.5 mm and up to 1.5 mm after lift-off, so we suggest that biofilm would be disrupted if the ultrasonic scaler tip is held 0.5 mm away from the surface to be cleaned.

To the best of our knowledge, we are the first to use high speed imaging combined with SLV to study cavitation around ultrasonic scalers. Previous studies using SCL to measure cavitation around ultrasonic scaler tips found minimal cavitation to be occurring at the end of the tip, and the low intensity of luminescence made it difficult to do further analysis to establish the extent of cavitation at the tip end [9,10]. The high speed images in this study confirm that substantial cavitation does occur at the end of ultrasonic scaler tips.

Previous work and this work confirms that cavitation occurs at the antinodes, or points of highest displacement, of ultrasonic scaler tips, with no cavitation occurring at the nodes [10]. In the literature, this was attributed to the higher amount of liquid being displaced by the scaler at the points where the amplitude of vibration is largest [10]. In the linear wave theory it can be shown that an antinode (node) for displacement is always an antinode (node) for pressure and vice versa. Therefore, the lowest pressure occurs at an antinode for displacement, which causes cavitation over there. As such, the antinodes are at the start of the cavitation cloud, not the centre (Fig 9).

As expected, the cavitation increased at higher power, with the area, width and height of the bubble cloud at the tip being larger at power 20 compared to power 10 for all three tips. This correlates with previously reported SCL results which also showed increased cavitation around ultrasonic scaler tips at higher power settings [3].

One limitation of this study is the fact that the scaler was submerged in a water tank, with the cooling water turned off, as this setup made high speed imaging easier. However, this is different to a clinical situation, where the scaler operates in air with cooling water flowing around the tip. Therefore the flow fields and possibly the cavitation patterns may be different clinically to what we have observed in this study. Therefore it would be useful to image the scaler with the cooling water turned on, or immersed in a water flow. The resolution of the imaging system with the 2x objective at 4x zoom was 5.6±0.1 μm. Therefore it could be possible that nano-sized bubbles were also generated but could not be detected. In addition, the water temperature in the container was found to increase by approximately 10°C after operating the scaler tip for 3 consecutive minutes. Further research is required to determine how temperature affects the amount of cavitation.

The image analysis method used in this work could be applied in a wide range of other studies for analysing bubbles or bubble cloud areas from high speed video frames. To obtain more accurate measurements of the width and height of the bubble cloud, the images could be straightened before performing the calculations (Fig 3). We found that tip 10P, the most pointed, produced the highest area, width and height of cavitation at the tip.

Tip 2, which is flat at the end, produced less cavitation at the tip. This could be because tip 10P has the highest displacement amplitude and tip 2 has the least displacement amplitude; the latter has a larger cross-section area. As the amplitude of pressure is proportional to the amplitude of the displacement (according to the linear wave theory), tip 10P is associated with the largest amplitude of pressure. Felver et al. [10] observed that, at a similar amplitude of displacement, tips from a similar piezoelectric system (EMS, Nyon, Switzerland) with a wide cross-section caused more cavitation. They hypothesised that tips with a wide cross-section would be able to displace more water compared to pointed tips. Further work is needed to understand how the cavitation around ultrasonic scaler tips is affected by tip shape, temperature, irrigant solution, cooling water flow and tip wear.

## Conclusion

This work has shown that cavitation does occur around the free end of ultrasonic scaler tips. The area of cavitation near the free end of the tips increases with power as well as with the amplitudes of displacement at the tips. The imaging and image analysis protocol developed in this study could be applied to other studies to quantitatively measure cavitation activity from high speed images. These results suggest that it is likely that cavitation from ultrasonic scalers could disrupt biofilm in a non-contact mode.

## Supporting Information

S1 Video High speed video of the bend of tip 1 at power 10 (refer to Fig 2 to see the part of the tip imaged), taken at 15,000 fps and played at 10 fps. (AVI)

S2 Video Individual cavitation bubbles on an ultrasonic scaler tip. High speed video of the inner part of the free end of tip 10P at power 10. Taken at 250,000 fps and played at 10 fps. Video continues from the frames shown in Fig 6. Individual microbubbles can be seen growing and collapsing.
(AVI)

S3 Video High speed video of tip 1 at maximum power. Cavitation clouds are seen occurring at the bend and at the free end of the tip. Video taken at 90,000 fps and played at 10 fps.
(AVI)

S4 Video High speed video of tip 10P at maximum power. Cavitation clouds are seen occurring at the bend and at the free end of the tip. Video taken at 90,000 fps and played at 10 fps.
(AVI)

S5 Video High speed video of tip 2 at maximum power. Cavitation clouds are seen occurring at the bend and at the free end of the tip. Video taken at 90,000 fps and played at 10 fps.
(AVI)
